# Peace Celebrations at King Edward VII. Hospital, Cardiff

**Published:** 1919-07-26

**Authors:** 


### Peace Celebrations at King Edward VII. Hospital, Cardiff.

THE Peace Celebrations at King Edward VII. Hospital, Cardiff, gave a very special pleasure to the patients. That was because the patients' wishes had been consulted, and their chief desire was to see their own friends a little longer than usual. On these lines the scheme of entertainment developed, with the result that when the patients' friends, numbering several hundreds, came to visit them on Saturday they remained all the afternoon. Tea was served to all the guests, each patient with his own little family party, and an excellent string band supplied appropriate music. It was intended that the band should be placed on a central lawn, but the heavy rain drove it into an empty ward, just emerging from the hands of the builders. This formed a rallying ground for all, where the King's mesage was read and a kind message from the Lord Mayor (who generously paid all the expenses of the entertainment). Here also the patients and their friends stood in silence for just a few seconds, in remembrance of the dead who could not be forgotten anywhere on that great day. Then someone raised the Welsh National Anthem, and emotion found its outlet in song. In the evening, after the patients' friends had left, there was a popular concert in the empty ward, whilst a quartette party made the rounds of all the wards so that every patient heard some of the music. Sir William James Thomas visited every patient during the afternoon. An outstanding feature of the day was the splendid devotion of the nurses and staff generally, who gave up their day entirely to the patients. It is hoped that they will have their "celebration" later.

